# Fc-Binding Cyclopeptide Induces Allostery from Fc to Fab: Revealed Through in Silico Structural Analysis to Anti-Phenobarbital Antibody

**DOI:** 10.3390/foods14081360

**Published:** 2025-04-15

**Authors:** Tao Zhou, Huiling Zhang, Xiaoting Yu, Kangliang Pan, Xiaojun Yao, Xing Shen, Hongtao Lei

**Affiliations:** 1 Guangdong Provincial Key Laboratory of Food Quality and Safety and Nation-Local Joint Engineering Research Center for Machining and Safety of Livestock and Poultry Products, South China Agricultural University, Guangzhou 510642, China; 2 College of Mathematics and Informatics & College of Software Engineering, South China Agricultural University, Guangzhou 510642, China; 3 Artificial Intelligence Drug Discovery Centre, College of Applied Sciences, Macao Polytechnic University, Macau 999078, China

**Keywords:** cyclopeptide, antibody, allostery, phenobarbital, food adulterant

## Abstract

Allostery is a fundamental biological phenomenon that occurs when a molecule binds to a protein’s allosteric site, triggering conformational changes that regulate the protein’s activity. However, allostery in antibodies remains largely unexplored, and only a few reports have focused on allostery from antigen-binding fragments (Fab) to crystallizable fragments (Fc). But this study, using anti-phenobarbital antibodies—which are widely applied for detecting the potential health food adulterant phenobarbital—as a model and employing multiple computational methods, is the first to identify a cyclopeptide (cyclo[Link-M-WFRHY-K]) that induces allostery from Fc to Fab in antibody and elucidates the underlying antibody allostery mechanism. The combination of molecular docking and multiple allosteric site prediction algorithms in these methods identified that the cyclopeptide binds to the interface of heavy chain region-1 (CH_1_) in antibody Fab and heavy chain region-2 (CH_2_) in antibody Fc. Meanwhile, molecular dynamics simulations combined with other analytical methods demonstrated that cyclopeptide induces global conformational shifts in the antibody, which ultimately alter the Fab domain and enhance its antigen-binding activity from Fc to Fab. This result will enable cyclopeptides as a potential Fab-targeted allosteric modulator to provide a new strategy for the regulation of antigen-binding activity and contribute to the construction of novel immunoassays for food safety and other applications using allosteric antibodies as the core technology. Furthermore, graph theory analysis further revealed a common allosteric signaling pathway within the antibody, involving residues Q123, S207, S326, C455, A558, Q778, D838, R975, R1102, P1146, V1200, and K1286, which will be very important for the engineering design of the anti-phenobarbital antibodies and other highly homologous antibodies. Finally, the non-covalent interaction analysis showed that allostery from Fc to Fab primarily involves residue signal transduction driven by hydrogen bonds and hydrophobic interactions.

## 1. Introduction

Allostery is a widespread intramolecular effect that plays a crucial role in regulating protein function and biological activity [1]. This effect involves ligand binding at distal allosteric sites through covalent modifications (e.g., nitration) or non-covalent interactions (e.g., ion coordination), triggering conformational rearrangements that precisely regulate functionality [2,3,4,5,6]. Owing to this property, allostery is involved in various biological processes, including signal transduction, enzyme catalysis, cell metabolism, and gene regulation [7]. Hence, it is often referred to as the “second secret of life”, following the genetic code [8]. The continuous development of structural and computational biology has led to the identification of an increasing number of allosteric proteins. Some researchers have boldly predicted that, except for fibrin, which lacks flexibility, allostery is a universal intrinsic property of most proteins [9]. However, as an immunoglobulin, studies on antibody allostery remain largely unexplored.

**Figure 1:**
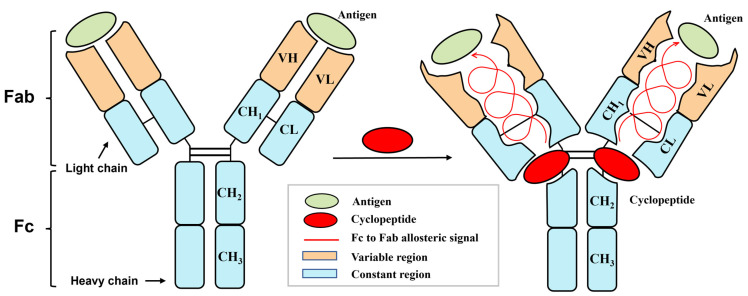
Schematic diagram of antibody structure and cyclopeptide-induced Fc to Fab (Fc→Fab) antibody allostery. Note: (i) Fab—fragment of antigen binding. (ii) Fc—crystallizable fragment. (iii) VH—variable heavy chain region. (iv) VL—variable light chain region. (v) CH—constant heavy chain region. (vi) CL, constant light chain region.

As a class of proteins with specific recognition characteristics for antigens, antibodies play a vital role in biomedical applications, such as immunoassays and precision therapy, and in food safety research as core technologies [10,11,12]. Classical theory holds that Immunoglobulin G (IgG) antibodies (Figure 1) are mediated by two domains, including the fragment of antigen binding (Fab) and the crystallizable fragment (Fc) [13]. The variable region of the Fab is the primary site for antigen binding, which determines recognition specificity, while the Fc binds to cell surface Fc receptors (FcRs) to mediate antibody-dependent cell-mediated cytotoxicity (ADCC) [14,15,16,17].

Traditionally, regulating antibody antigen specificity is primarily focused on the variable region of Fab (e.g., antigen design, molecular modification) [13], which has limitations, including complexity and high cost. But allostery in protein offers novel opportunities for regulating antibody Fab activity. However, as mentioned above, current research on antibody allostery is still limited and primarily focuses on antibody allostery from antigen-binding fragments (Fab) to crystallizable fragments (Fc), where the Fab variable region changes the Fc functional activity under covalent or non-covalent interaction modification [13]. For instance, Sun et al. [18] found that antigen modification enhances crystallizable fragment–Fc receptor (Fc-FcR) affinity, Xin et al. [19] found that amino acid differences in Fab affect Fc activity, and Zhao et al. [20] demonstrated that antigen allostery strengthens interactions between the Fc and Fc receptor. However, whether the Fc region can regulate Fab functional activity through simple ligand binding remains unknown. To date, the concept of allostery from Fc to Fab in antibodies has not been explored.

Phenobarbital is a sedative commonly used to treat epilepsy and anxiety. However, excessive intake can lead to severe adverse effects, including respiratory depression and an increased risk of cancer [21]. Additionally, it is prone to adulteration in traditional Chinese medicinal products, such as quick-acting herbal pills. Consequently, China has classified it as a prohibited substance in health foods and has established corresponding detection standards and analytical methods [22]. Among these methods, anti-phenobarbital antibodies serve as the core technology for developing immunoassay-based phenobarbital detection [23]. As one of the earliest studied antibody types, it provides crucial reference and a readily available template for designing other antibodies.

This study utilized an anti-phenobarbital antibody as a model and integrated multiple computational approaches to first identify an Fc-binding cyclopeptide (cyclo[Link-M-WFRHY-K]). This cyclopeptide induces conformational transitions from Fc to Fab, thereby improving antigen-binding activity in the Fab domain. To investigate the mechanism of antibody allostery from Fc to Fab induced by cyclopeptide, we first identified the cyclopeptide’s binding site (allosteric site) on the Fc region of antibody. This was achieved by integrating molecular docking and multiple allosteric site prediction algorithms. Based on the docking results and control experiments, we established six antibody complex simulation systems incorporating endogenous glycans, antigens, and cyclopeptides. Integrated analysis of structural fluctuations, conformational changes, and residue correlation revealed allostery from Fc to Fab that influences the Fab domain and antigen binding activity by the cyclopeptide. Furthermore, graph theoretical analysis uncovered a common allosteric pathway across the antibody architectures. Comprehensive non-covalent interaction analysis delineated the core drivers governing allosteric signal initiation, propagation, and functional output in antibody systems. These studies will establish a new paradigm for antibody allostery from Fc to Fab and offer novel insights into immunoassays for the analysis of food safety.

## 2. Materials and Methods

In this study, the anti-phenobarbital antibody (PDBID: 1IGY) structure was obtained from the RCSB Protein Database [24], while the antigen (phenobarbital) and cyclopeptide (cyclo[Link-M-WFRHY-K]) structures were, respectively, obtained from the PubChem database and Menegatti et al. [25].

### 2.1. Molecular Docking

To identify potential allosteric sites on the antibody, we first applied various predictive algorithms to the anti-phenobarbital Immunoglobulin G-1 (IgG_1_) antibody, including Passer [26], CavityPlus [27], and Allosite [28] from the ASD database. We then used the AutoDock Vina 1.2.0 docking program to validate the predicted allosteric sites [29]. By aligning the predicted allosteric and ligand binding sites, we defined the docking box size and position along the XYZ axes to obtain docking results. The docking boxes for cyclopeptide–antibody interactions at different predicted sites were defined based on their center coordinates (in nm) within the spatial coordinate system: (i) (1.0, −2.8, 6.6), (ii) (−0.2, −1.9, 6.6), (iii) (−0.8, 0.8, 2.6), and (iv) (1.9, 2.0, 4.4). Meanwhile, the docking box for the allosteric site was uniformly defined as a cubic box with dimensions of 3.08 × 3.08 × 3.08 nm. The default scoring function of the docking software evaluated the binding strength between the antibody and cyclopeptide at predicted site. Visualization of cyclopeptide–antibody docking complexes at different sites in Discovery Studio Visualizer 2019 software confirmed the validity of the allosteric sites [30]. To ensure experimental completeness, the antigen substrate and endogenous glycans were molecularly docked with the antibody Fab and Fc, respectively, according to the docking boxes of the same size mentioned above. The ligand–antibody complex structures (cyclopeptide, antigen substrate, and endogenous glycans) generated by molecular docking will serve as input files for molecular dynamics simulations.

### 2.2. System Construction and Molecular Dynamics Simulations

Based on molecular docking results, the optimal binding site and conformation of the ligand–antibody complex (cyclopeptide, antigen, and glycans) were identified. Six simulation systems were then constructed: (i) non-glycosylated antibody; (ii) non-glycosylated antibody–cyclopeptide; (iii) glycosylated antibody; (iv) glycosylated antibody–cyclopeptide; (v) glycosylated antibody–antigen; (vi) glycosylated antibody–cyclopeptide–antigen. Molecular dynamics simulations were then conducted using GROMACS 2021.3 [31].

The Amber-99SB-ILDN force field was chosen for antibody topology parameterization due to its strong performance in modeling protein and nucleic acid systems. GAFF force fields and topology parameters for glycans, cyclopeptides, and antigens were generated using the Antechamber Python script in AmberTools 23 [32,33,34,35], ensuring compatibility with Amber force fields and accurate ligand description. Due to the higher dynamic flexibility of the antigen compared to the cyclopeptide and glycans, the Restrained Electrostatic Potential (RESP) method was used to calculate antigen charges, while Bond Charge Correction (BCC) charges were assigned to glycans and cyclopeptide.

System construction followed the GROMACS official tutorial for protein–ligand complex setup in molecular dynamics simulations. Complex systems were placed in cubic simulation boxes with a minimum boundary of 1 nm in each XYZ direction to ensure sufficient spacing. Meanwhile, each antibody complex system was built in a simulation box at the central coordinate position of the antibody structure (−0.5, −0.5, 4.2), and the box size was 16.94 × 16.94 × 16.94 nm. TIP3P water molecules were introduced to mimic the solvent environment of antibody–ligand complexes. Sodium or chloride ions were added to neutralize the systems, ensuring an electrically neutral environment that closely mimics physiological conditions. Energy minimization was performed using the steepest descent (SD) and conjugate gradient (CG) algorithms to remove unfavorable atomic interactions. The systems were then equilibrated under constant temperature and pressure (NPT) using a velocity rescale thermostat and a Berendsen barostat. The LINCS algorithm was used to constrain hydrogen bonds, refining complex–water interactions and preventing excessive interactions caused by harmonic potentials. Short-range non-bonded interactions and van der Waals forces were computed with a 10 Å cutoff, while electrostatic interactions were calculated using the Particle Mesh Ewald (PME) algorithm. Each system was simulated for 300 ns, with trajectories recorded every 10 ps for subsequent analysis.

### 2.3. Analytical Metrics

#### 2.3.1. Analysis of Antibody Backbone Structural Fluctuation (RMSD, RMSF, Rg)

The “rms”, “rmsf”, and “gyrate” modules provided by GROMACS 2021.3 software were used to obtain the above-mentioned antibody backbone and structural fluctuation parameters (root mean square deviation (RMSD), radius of gyration (Rg), and root mean square fluctuation (RMSF)) from the molecular dynamics simulation systems of each antibody complex.

#### 2.3.2. Analysis of Antibody Dynamic Conformational Ensembles (PCA and FEL)

The bio3d 2.5 [36] package in the R platform was used to select protein Cα atoms for structural alignment based on the initial structure of the antibody and the trajectory after removing translation and rotational motion, so as to intuitively analyze the principal component analysis (PCA) of the antibody conformational ensemble [37,38,39]. Additionally, the “gmx covar” command was used in conjunction with the “gmx aneig” command module to calculate the characteristic eigenvectors (covariance matrix) and eigenvalues of Cα atoms in the antibody trajectories based on Cartesian coordinates. FEL maps were constructed based on the proportions of PC in each system. The trajectory was projected onto the primary component plot of the eigenvectors, and then free energy landscape (FEL) was plotted using PC1 and PC2, where the free energy is expressed as

G = −kT + lnP(1)

Here, k is the Boltzmann constant, T is the simulation system temperature, and P represents the density of probabilities for various conformation [40,41,42].

#### 2.3.3. Analysis of Antibody Residue Correlation (DCCM and RCM)

The covariance matrix was obtained from the translationally and rotationally eliminated trajectories and the initial antibody structure using the “gmx covar” command, and the dynamic cross-correlation matrix (DCCM) of the motions between the Cα atoms of the residue backbone was calculated based on the covariance information in this matrix [43,44,45,46]. The calculation of covariance is given by

(2) $$c(i,j)=<∆r_{i}.∆r_{j}>$$

where ∆r represents the displacement vector of atoms, i and j represent different residues, and the normalized covariance (cross-correlation) calculation is

(3) $$C(i,j)=\frac{c(i,j)}{{\left[c(i,i)c(i,j)\right]}^{1/2}}=\frac{<∆r_{i}.∆r_{j}>.}{<∆r_{i}^{2}{>}^{1/2}.<∆r_{j}^{2}{>}^{1/2}}$$

The thickness of the diagonal in the matrix represents changes in the α-helix structures of the four antibody peptide chains (heavy chain and light chain), while lines parallel or orthogonal to the diagonal represent β-sheet structures. RCM was obtained by using the “gmx mdmat” command module combined with ConAn [47] residue contact analysis software, based on the antibody structure and trajectory, by selecting the residue range and defining the cutoff distance for residue contacts [48,49].

#### 2.3.4. Analysis of the Allosteric Transduction Pathway Within Antibody

We employed the residue distance cross-correlation matrix (RDCM) to investigate the signaling mechanisms triggered by conformational change. RDCM is a weighted node matrix formed by the fusion of RCM and DCCM, where nodes represent residues and edge weights correspond to matrix values. This approach provides a RDCM-based representation of the antibody’s conformational signaling network. Using the Dijkstra shortest path algorithm, we identified the shortest signal transmission pathways by traversing all nodes (residues) within the matrix network. After normalizing the relevant edges, significant contribution residues (based on a defined threshold) were extracted using the shortest path graph (SPM) script developed by Sílvia Osuna’s team and mapped onto the antibody structure to identify the allosteric transduction pathways for each system [50,51].

#### 2.3.5. Analysis of Non-Covalent Interactions Within Antibody

We combined various force analysis modules in the MDanalysis [52] software program with the DuIvyProcedures 1.0.0 [53] analysis tool to analyze and visualize the temporal changes in different non-covalent interactions (such as hydrogen bonding interactions, hydrophobic interactions,π–cation interactions, π–π interactions, etc.) occurring at the antibody–antigen binding site, antibody–cyclopeptide binding site, and throughout the entire antibody structure. In this analysis, the existence of hydrogen-bonding interactions was determined based on the following geometric criteria: the distance between the hydrogen-donating atom (donor) and the hydrogen-accepting atom (acceptor) within adjacent residues or between a residue and a ligand was less than 3 Å, and the angle formed by the hydrogen donor–acceptor was greater than 150 degrees [54,55]. Furthermore, we considered the existence of hydrophobic interactions if the distance between Cα atoms of adjacent residues was less than 4 Å [56]. Finally, the following criteria were employed to define electrostatic interactions: Cation–π interactions were deemed to be present when the distance between aromatic ring atoms and positively charged nitrogen atoms in adjacent residues (or residue–ligand) was less than 6 Å. Meanwhile, π–π interactions were considered to exist when the distance between the centroids of aromatic ring functional groups in neighboring residues was less than 5 Å [57].

## 3. Results and Discussion

### 3.1. Identification of Antibody Allosteric Site

**Figure 2:**
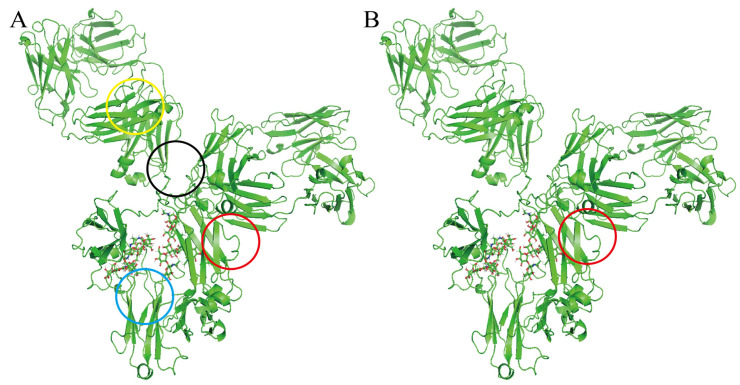
Allosteric site algorithm and molecular docking results: (**A**) cyclopeptide–antibody binding site predicted by allosteric site algorithm; (**B**) cyclopeptide–antibody binding site validated by molecular docking. Note: (i) the lower, yellow-marked region of the Fab domain’s variable region; (ii) the black-marked region of the upper hinge region; (iii) the blue-marked region between the Fc domain’s heavy chain constant regions-2 (CH_2_) and -3 (CH_3_); (iv) the red-marked region between the Fab domain’s heavy chain constant regions-1 (CH_1_) and the Fc domain’s heavy chain constant region-2 (CH_2_).

The accurate prediction of an allosteric site represents a critical step in elucidating antibody allosteric mechanisms. In this study, we define the ligand-binding region of the antibody structure other than the Fab active site as the potential allosteric site of the antibody. Integration of antibody structural analysis with multiple prediction algorithms identified more than a dozen potential allosteric sites. Computational predictions localized four major allosteric hotspots (Figure 2A).

**Figure 3:**
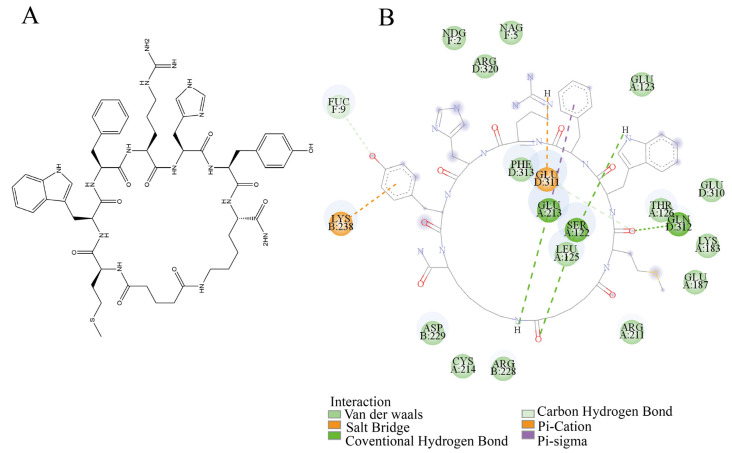
Interaction of cyclopeptide and antibody: (**A**) Structure of cyclopeptide. (**B**) Cyclopeptide bindings site (the interface between CH_1_ of the Fab domain and CH_2_ of the Fc domain).

The binding sites were further identified using a molecular docking model of the cyclopeptide–antibody system. The cyclopeptide (Figure 3A) exhibited a higher docking affinity (−7.124 kcal/mol) at the interface between CH_1_ of the Fab domain and CH_2_ of the Fc domain (red region in Figure 2B). Other predicted allosteric sites (red-marked outer regions in Figure 2A) exhibited an average docking affinity of −5 to −6 kcal/mol. Furthermore, we analyzed the docking interactions at the CH_1_-CH_2_ interface (Figure 3B); the cyclopeptide’s benzene ring, indole ring, and hydroxyl group formed conventional hydrogen bonds and van der Waals interactions with C213, L125, and E122 of light chain A and F312 of heavy chain D in antibody. Additionally, P238 of heavy chain B and Q311 of heavy chain D interacted with the guanidinium and phenolic benzene rings of the cyclopeptide’s side chain via π–cation and salt bridge interactions. Meanwhile, C213 and F311 also participate in weak interactions with the cyclopeptide, including carbon–hydrogen bonding and π–sigma. The abundance of functional residues and non-covalent interactions at this site, compared to other predicted sites, suggests that the CH_1_-CH_2_ interface serves as the key allosteric site where the cyclopeptide binds and induces antibody allostery from Fc to Fab. This finding also provides theoretical support for the precise localization of the Fc fragment in the cyclopeptide–antibody complex, as reported by Menegatti et al. [25].

### 3.2. Influence of Cyclopeptide on Antibody Backbone Structure

To investigate the influence of cyclopeptide on the overall antibody structural stability from the perspective of the antibody backbone structure, we calculated the root mean square deviation (RMSD), radius of gyration (Rg), and root mean square fluctuation (RMSF) of the antibody in each complex system. Moreover, the study by Ikeuchi et al. demonstrated that a moderate balance between structural stability and flexibility of antibodies contributes to improved antigen-binding affinity [58].

**Figure 4:**
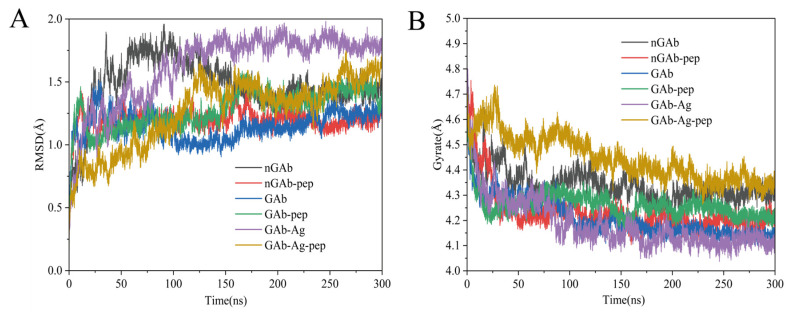
Root mean square deviation (RMSD) curves (**A**) and radius of gyration (Rg) curves (**B**) of all antibody complex systems. Note: (i) nGAb (black)—non-glycosylated antibody system. (ii) nGab-pep (red)—non-glycosylated antibody cyclopeptide system. (iii) Gab (blue)—glycosylated antibody system. (iv) Gab-pep (green)—glycosylated antibody cyclopeptide system. (v) Gab-Ag (purple)—glycosylated antibody antigen system. (vi) Gab-Ag-pep (yellow)—glycosylated antibody cyclopeptide antigen system.

Root mean square deviation (RMSD) measures the deviation of the protein backbone from its initial conformation and serves as a key indicator of whether the system has reached dynamic equilibrium, as well as the extent to which cyclopeptides influence antibody structure stability [59]. The temporal variations in antibody RMSD are shown in Figure 4A. The curve trend comparison between systems with (red, green, and yellow curves) and without cyclopeptides (black, blue, and purple curves) found that cyclopeptides markedly alter the antibody RMSD value. Meanwhile, cyclopeptides influenced the time required for each system to reach dynamic equilibrium. These findings suggest that the cyclopeptide induces global structural changes in the antibody, progressively transmitting from the Fc to the Fab region, thereby indirectly altering the Fab domain and its structural stability, ultimately leading to improved antigen binding. In addition, comparing the RMSD curves of antibody in complex systems with and without endogenous glycans (black and blue curves) shows that endogenous glycans reduce RMSD fluctuations, confirming glycan’s stabilizing effect, as reported by Pawlowski et al. [60]. Furthermore, comparing the RMSD curves of antibody in complex systems with and without antigen (blue and purple curves) shows that antigen binding increases RMSD, consistent with Zhao et al.’s [20] description of antigen-induced allostery.

As a powerful tool to describe the compactness of proteins in solution, radius of gyration (Rg) is another important parameter for assessing the structural stability of antibodies [61]. As illustrated in Figure 4B, the antibody Rg values increase with cyclopeptide binding (red, green, and yellow curves) compared to those without cyclopeptides (black, blue, and purple curves), suggesting a more relaxed antibody structure. The relaxed structure further indirectly changes the stability of the Fab domain’s structure from Fc to Fab in the form of overall structural changes, consistent the results in RMSD.

**Figure 5:**
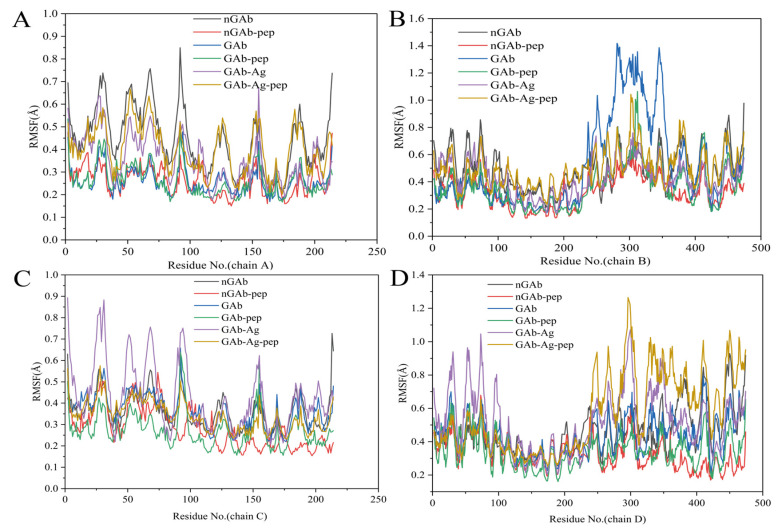
Root mean square fluctuation (RMSF) data for the four peptide chains (two heavy and two light chains) of all antibody complex systems. (**A**) RMSF of light chain A. (**B**) RMSF of heavy chain B. (**C**) RMSF of light chain C. (**D**) RMSF of heavy chain D. Note: (i) nGAb (black)—non-glycosylated antibody system. (ii) nGab-pep (red)—non-glycosylated antibody cyclopeptide system. (iii) Gab (blue)—glycosylated antibody system. (iv) Gab-pep (green)—glycosylated antibody cyclopeptide system. (v) Gab-Ag (purple)—glycosylated antibody antigen system. (vi) Gab-Ag-pep (yellow)—glycosylated antibody cyclopeptide antigen system.

Root mean square fluctuation (RMSF) quantifies the average fluctuation amplitude of residues in each antibody chain and serves as a crucial indicator of ligand binding specificity and its impact on flexibility [62]. Figure 5 depicts the RMSF profiles of four antibody chains (light chains A and C; heavy chains B and D). Comparing RMSF fluctuations of antibody in the systems with and without cyclopeptides (Figure 5A–D) shows that the cyclopeptide alters the RMSF values of the four antibody chains in each system. This indicates that the cyclopeptide has a clear overall impact on the antibody structure. In addition, the RMSF fluctuation trend of the Fab region (residues 0–214 of the light and heavy chains) differs between the system with cyclopeptides (red, green, and yellow curves) and the system without cyclopeptides (black, blue, and purple curves). This phenomenon indicates that the cyclopeptide exerts an Fc to Fab allosteric effect on the Fab domain and its structural stability in both antigen-bound and unbound states, thereby promoting enhanced antigen-binding activity. Similarly, the RMSF fluctuation trend in the Fc region (residues 214–400 of the heavy chain) shows distinct differences between systems with cyclopeptide (red, green, and yellow curves) and those without (black, blue, and purple curves), demonstrating the effective binding of cyclopeptide to Fc and it allosteric influence on its structure.

Therefore, the backbone structural parameters—including root mean RMSF, RMSD, and Rg—on the antibody suggest that cyclopeptides could serve as a novel Fab positive allosteric modulator for immunoassay methods centered on antigen–antibody interactions. Additionally, residues with high RMSF fluctuation values may offer critical insights for optimizing the design of anti-phenobarbital antibodies.

### 3.3. Influence of Cyclopeptide on Antibody Dynamic Conformational Ensembles

To clarify the impact of cyclopeptide on antibody dynamic conformational ensembles, principal component analysis (PCA) and free energy landscape (FEL) analysis were conducted on the frame-by-frame conformations of antibodies in each complex system throughout the entire trajectory. Moreover, the study by Ma et al. revealed that relatively compact and low-energy protein conformational ensembles facilitate effective binding between the protein and ligand at the orthosteric site [63]. Therefore, the analysis of conformation ensemble parameters will closely align with the previous structural parameter results, providing strong evidence for cyclopeptide-induced allosteric modulation of antibodies from Fc to Fab, which enhances antigen-binding activity. This also lays a theoretical foundation for the innovation of immune detection methods based on antigen–antibody specificity.

**Figure 6:**
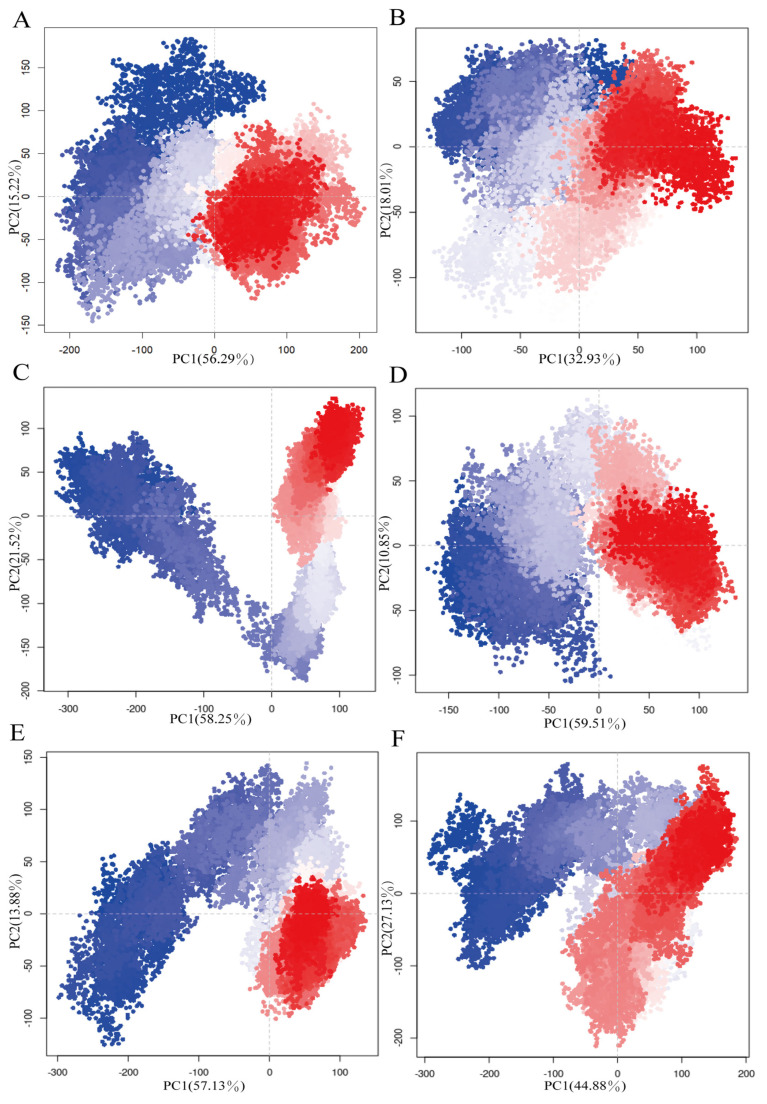
Principal component analysis (PCA) of dynamic conformations in all antibody systems. (**A**) Non-glycosylated antibody system. (**B**) Non-glycosylated antibody–cyclopeptide system. (**C**) Glycosylated antibody system. (**D**) Glycosylated antibody–cyclopeptide system. (**E**) Glycosylated antibody–antigen system. (**F**) Glycosylated antibody–cyclopeptide–antigen system.

Principal component analysis (PCA) clusters similar antibody structures throughout the molecular dynamics trajectory and serves as a key indicator of antibody conformational changes. As shown in Figure 6, the cumulative proportions of the first two principal components (PCs) in the single antibody system with cyclopeptides are 50.94% (Figure 6B) and 70.36% (Figure 6D), respectively. These values are notably lower than those of the non-glycosylated (71.51%, Figure 6A) and glycosylated antibody systems without cyclopeptides (79.5%, Figure 6C). These findings suggest that cyclopeptides decrease structural clustering in antibodies, significantly altering their conformation and ultimately affecting fab domain. Similarly, the cumulative proportions of the first two principal components (PCs) in the antigen-bound (71.01%, Figure 6E) and glycosylated antibody systems (79.5%, Figure 6C) highlight the impact of antigen binding on antibody structural aggregation, demonstrating that antigen binding induces conformational changes, in line with the findings of Zhao et al. [20]. In contrast, in the antigen–cyclopeptide binding antibody system (72.01%, Figure 6F), the cumulative proportions of the first two PCs were similar to those observed in the single antigen- or cyclopeptide-binding systems. This suggests that the cyclopeptide counteracts the antigen-induced allostery through an inhibition mechanism from Fc to Fab, thereby promoting more compact conformational ensembles in the Fab domain and enhancing its antigen-binding ability.

**Figure 7:**
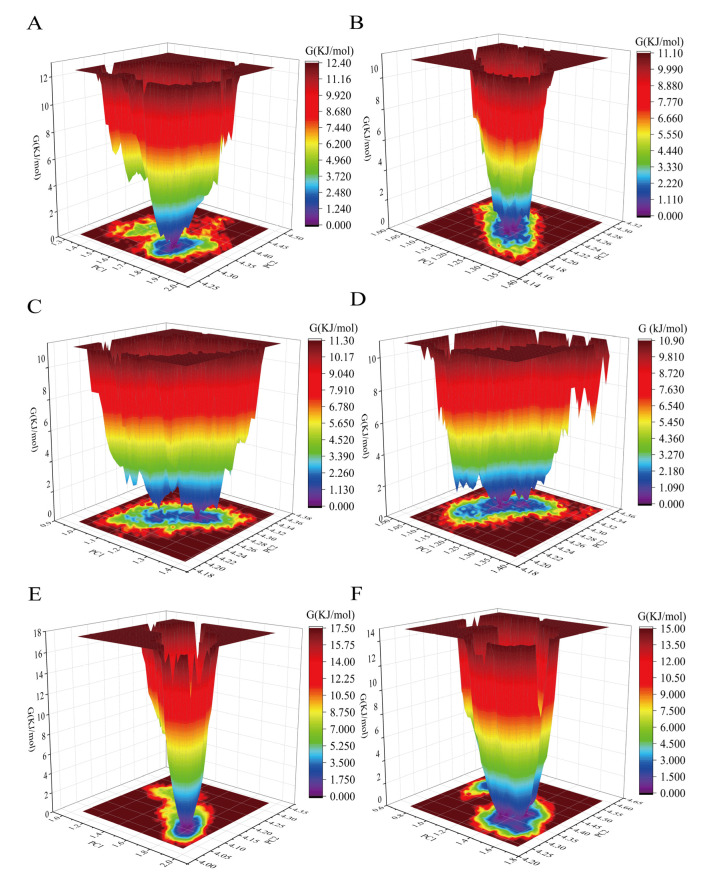
Free energy landscape (FEL) of dynamic conformations in all antibody systems. (**A**) Non-glycosylated antibody system. (**B**) Non-glycosylated antibody–cyclopeptide system. (**C**) Glycosylated antibody system. (**D**) Glycosylated antibody–cyclopeptide system. (**E**) Glycosylated antibody–antigen system. (**F**) Glycosylated antibody–cyclopeptide–antigen system.

Free energy landscape (FEL) is a crucial metric for assessing antibody conformational changes from an energy perspective. As shown in Figure 7, the single antibody system bound to the cyclopeptide (Figure 7B,D) exhibits more metastable, high-energy conformations than the unbound system (Figure 7A,C). This suggests that cyclopeptide binding induces a high-energy, unstable conformational state in the antibody, which significantly affects the Fab domain in manner from Fc to Fab, consistent with the PCA results. In the antigen-binding system (Figure 7E), antigen binding was found to increase the distribution of high-energy antibody conformations. This further supports the findings of Zhao et al. regarding the allostery of the antigen. However, the increase in metastable conformational states in the cyclopeptide–antigen-binding system (Figure 7F) suggests that cyclopeptide binding counteracts the antigen-induced allostery from Fc to Fab, ultimately promoting the transition of low-energy conformational ensembles in the Fab domain and enhancing antigen binding in a manner consistent with the trends observed in the PCA results.

### 3.4. Influence of Cyclopeptide on Antibody Residue Correlation

**Figure 8:**
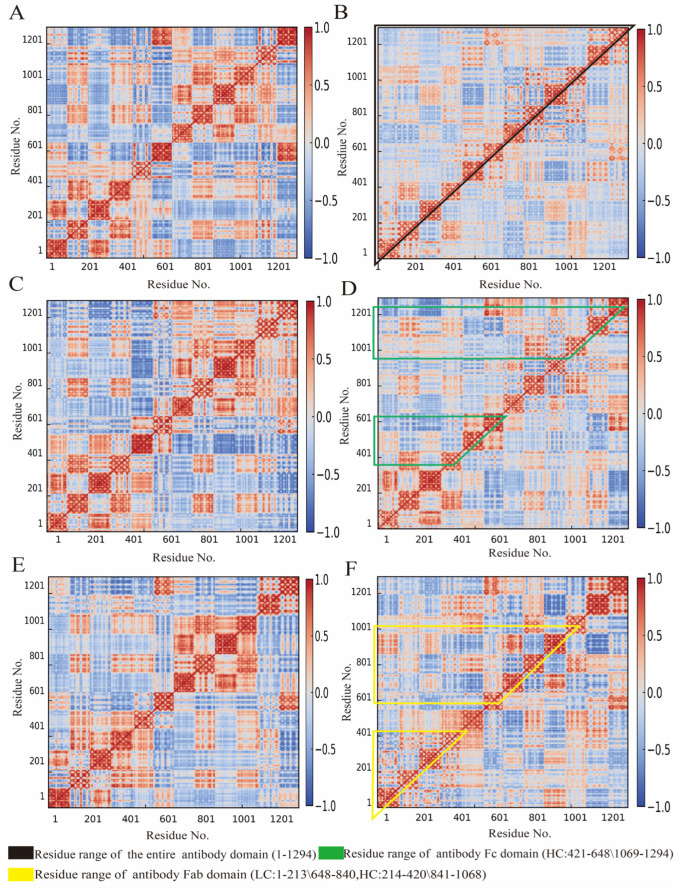
Dynamic cross-correlation matrix (DCCM) of antibody residues in all systems. (**A**) Non-glycosylated antibody system. (**B**) Non-glycosylated antibody–cyclopeptide system. (**C**) Glycosylated antibody system. (**D**) Glycosylated antibody–cyclopeptide system. (**E**) Glycosylated antibody–antigen system. (**F**) Glycosylated antibody–cyclopeptide–antigen system.

A dynamic cross-correlation matrix (DCCM) is a powerful tool for describing the dynamic motions within antibody structures and between residues, providing deeper insights into antibody allostery. As shown in Figure 8, the DCCM represents highly positively correlated regions in red and negatively correlated regions in blue. The color intensity indicates the degree of correlated motion between antibody residues. Black-marked regions denote the overall antibody structure, while green represents Fc domain residues (including both left and right heavy chain Fc ends) and yellow represents Fab domain residues (covering both left and right light chains as well as heavy chain Fab ends). The data indicate that in both glycan-containing and glycan-free systems (Figure 8A,C), cyclopeptides induce negatively correlated motion changes (blue) in the entire antibody structure and Fc domain (Figure 8B,D). Furthermore, Fab also exhibits noticeable negative correlated changes (Figure 8E,F). These results suggest that: (i) cyclopeptides bind effectively to antibody and induce allostery; (ii) cyclopeptides also inhibit antigen-induced conformational changes, thereby promoting intradomain residue correlation movements within the antibody Fab region and enhancing antigen recognition capability. Moreover, our structural analysis corroborates the stabilizing role of glycans and the allostery of antigens, as reported by Pawlowski et al. [60] and Zhao et al. [20].

**Figure 9:**
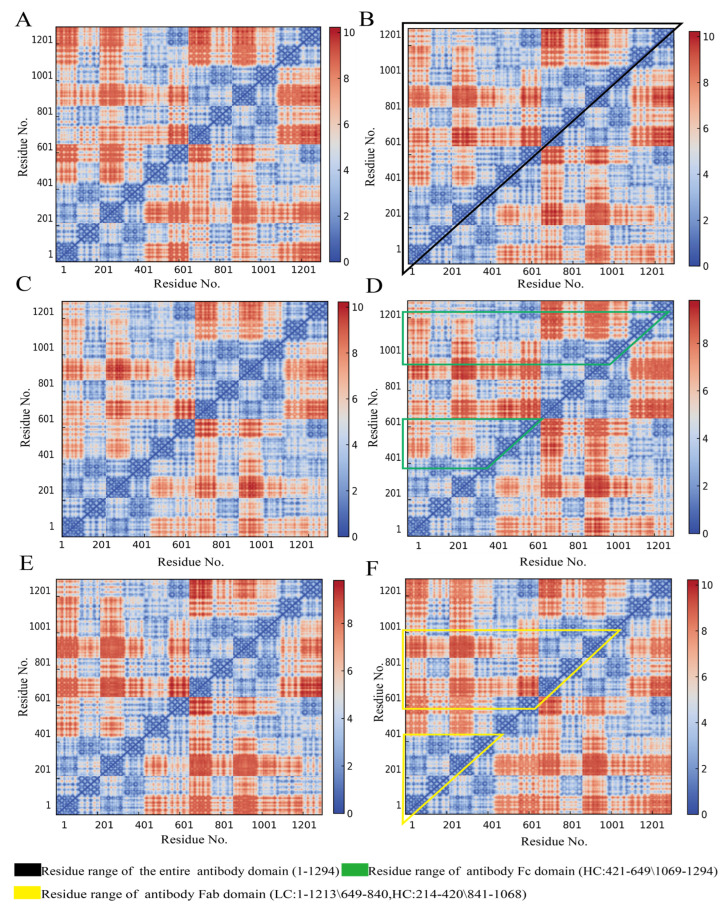
Residue contact matrix (RCM) of antibody residues in all systems. (**A**) Non-glycosylated antibody system. (**B**) Non-glycosylated antibody–cyclopeptide system. (**C**) Glycosylated antibody system. (**D**) Glycosylated antibody–cyclopeptide system. (**E**) Glycosylated antibody–antigen system. (**F**) Glycosylated antibody–cyclopeptide–antigen system.

The residue contact matrix (RCM) encodes residue distance information based on predefined residue ranges and distance cutoffs, capturing structural dynamics within antibodies. Compared to a DCCM based on atomic coordinates, the RCM emphasizes the correlation in residue contact distances and provides a clearer representation of antibody structural changes. As shown in Figure 9, the α-helix structures of the four main chains in the antibody exhibit minimal changes in RCM across different systems. The irregular orientation of antibody chains prevents the formation of a complete β-sheet structure; instead, the chains are connected by curved peptide segments. The black-bordered sections illustrate overall structural changes, showing that in antibody systems with and without glycans (Figure 9A,C), cyclopeptide addition induces residue distance changes similar to those observed in DCCM, though to a lesser extent (Figure 9B,D). Additionally, Fab (yellow border) and Fc (green border) region distances were compared in the presence and absence of cyclopeptides. The cyclopeptide significantly reduces the distance between residues in the antigen-binding region through allostery from Fc to Fab, distinct from that induced by the antigen, thus promoting stronger correlation interactions and tighter antigen-binding activity (Figure 9E,F).

DCCM and RCM analyses revealed that the cyclopeptide promotes the overall antibody structure and residue correlations within the Fab region. According to the relevant study by Chong et al., enhanced residue correlation serves as a direct indicator of increased binding affinity between the antibody Fab domain and the ligand antigen [64]. Additionally, compared with the indirect effects observed in previous structural parameter analyses, such residue correlations more directly characterize the intramolecular structural changes in the antibody induced by cyclopeptide binding. Collectively, these findings, in conjunction with previous analyses of conformational ensembles and backbone structural parameters, provide further validation of the cyclopeptide-induced Fc to Fab allosteric modulation theory and suggest promising avenues for innovatively regulating antigen–antibody binding interactions.

### 3.5. Allosteric Pathways of Antibody

The allosteric mechanism of antibodies is closely associated with signal transduction. To investigate this transmission mechanism, we identified allosteric pathways in antibodies by analyzing key hotspot residues across antibodies from different complex systems. This was achieved using the residue distance correlation matrix (RDCM) in combination with the Dijkstra shortest path and shortest path graph (SPM) algorithms. The RDCM integrates the dynamic cross-correlation matrix (DCCM) and the residue contact matrix (RCM). These key residues were identified by iteratively parsing high-weight threshold nodes from the RDCM fusion matrix using the Dijkstra algorithm combined with the SPM method, which exhibit significantly higher weight thresholds compared to other residue nodes. Simultaneously, to clarify the sequential arrangement of the residues, we implemented a systematic renumbering scheme for the antibody’s four polypeptide chains, following an alternating light–heavy chain pattern (L-H-L’-H’) to reflect its characteristic domain organization.

**Figure 10:**
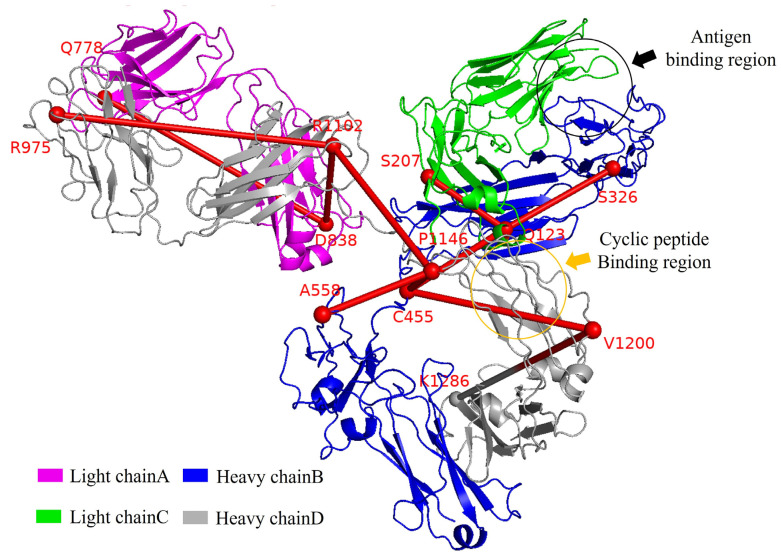
Schematic representation of antibody allosteric pathway. The red line indicates the allosteric pathway spanning the antibody structure. Binding sites for the cyclopeptide and antigen substrate are highlighted in yellow and black, respectively.

The statistical results are presented in Table 1. We found that all allosteric pathways pass through the key binding regions of antibody, including the Fab, Fc, and hinge regions, with the hinge region serving as the central hub of transmission. Moreover, the introduction of the cyclopeptide altered the key residues involved in the allosteric pathways of antibody in these systems. By analyzing the common residues across antibody of all systems conformational pathways, we identified an intramolecular signaling pathway within the antibody (Figure 10), comprising residues Q123, S207, S326, C455, A558, Q778, D838, R975, R1102, P1146, V1200, and K1286. This pathway is the primary route through which cyclopeptides exert allostery on the entire antibody structure and influence the Fab domain’s structure. Additionally, we found that the hotspot residues in this pathway are closely associated with functional residues in the cyclopeptide binding site, such as residue Q123, which was adjacent to residues S122 and L125. This indicates that Q123 is a key site for the transmission of the cyclopeptide’s allostery to the Fab domain.

Additionally, we observed that each functional domain of the antibody (Fab and Fc) increases the density of hotspot residues in the pathway due to the separate binding of antigen substrates, endogenous glycans, and the cyclopeptide. However, when all three ligands bind simultaneously, the residue distribution in the pathway reaches a delicate balance. These findings indicate that cyclopeptide binding produces intramolecular effects opposite to those of endogenous glycans and antigen substrates. Interestingly, we found that the signaling pathways involved with cyclopeptides not only display hotspot residues near the allosteric site but also at the lower end of the Fc domain. This suggests that cyclopeptides may exert potential allostery on Fc-Fc receptor interactions.

Therefore, this study on the antibody allosteric pathway, using the anti-phenobarbital antibody as a model, enhances the understanding of allostery theory from Fc to Fab and provides novel insights into the allostery-based antibody engineering design of anti-phenobarbital and its homologous antibodies.

### 3.6. Key Drivers of Antibody Allostery

To elucidate the key drivers of antibody allostery from Fc to Fab, we calculated the non-covalent interaction occupancy of the antibody throughout the entire simulation for three key aspects: the origin, transmission, and final impact of allostery. As shown in Table 2, the occupancy of hydrogen bond interactions in the antigen-binding region (orthosteric site) was 16.7% with cyclopeptide binding and 4.5% without. The hydrophobic interaction occupancy was 22.2% with cyclopeptide binding and 23.3% without. Meanwhile, the occupancy of hydrogen bond interactions in the cyclopeptide binding region (allosteric site) was 17.5% with antigen binding and 67.1% without. The hydrophobic interaction occupancy was 35.8% with antigen binding and 46.8% without. These changes suggest that cyclopeptide binding stabilizes non-covalent interactions at the antigen binding site and enhances antigen binding. The allostery from Fc to Fab is typically bidirectional, and antigen binding also promotes the binding of the cyclopeptide to the antibody. Additionally, the statistics of non-covalent interactions across the overall antibody structure in each system (Table 3) show that hydrogen bonding (200–220) and hydrophobic interactions (40,500–41,300) are the primary driving forces behind allosteric transmission mediated by cyclopeptide binding.

In the analysis of electrostatic interactions, it was found that the antigen lacks an aromatic ring structure, which prevents the formation of stable π–π and π–cation interactions. Moreover, the cyclopeptide-binding region near the hinge of the Fc domain lacks stable π interactions and salt bridges due to the irregular bending of the hinge region. Analysis of the temporal occupancy of electrostatic interactions across the overall antibody structure in each complex system revealed no significant change, suggesting that electrostatic interactions are not the primary drivers of the allostery.

Briefly, hydrogen bonding and hydrophobic interactions are the primary drivers of the three allosteric processes (initiation, transmission, and final impact) in cyclopeptide-induced antibody allostery.

## 4. Conclusions

In this study, using the antibody against phenobarbital as the model, antibody allostery from Fc to Fab induced by the cyclopeptide (cyclo[Link-M-WFRHY-K]) was investigated through in silico analysis for the first time: (i) The cyclopeptide binding site (allosteric site) is located between the CH1 region of the Fab domain and the CH2 region of the Fc domain in the antibody to phenobarbital. (ii) Cyclopeptide binding induces progressive changes in the antibody backbone, overall conformation, and residue correlations, leading to structural instability from Fc to Fab, which may influence Fab domain function and antigen binding. (iii) A conserved allosteric signaling pathway was identified within the antibody, particularly spanning residues Q123, S207, S326, C455, A558, Q778, D838, R975, R1102, P1146, V1200, and K128. (iv) The core mechanism of allostery from Fc to Fab in antibodies is governed by residue interactions primarily driven by hydrophobic and hydrogen bonding.

In summary, this study provides a valuable approach for designing Fab-targeted allosteric modulators. It also offers a novel strategy for modulating antigen-binding activity within the Fab domain. Future research could build on these findings through experimental validation to establish allosteric immunoassay methods for applications in food safety, environmental monitoring, therapeutic analysis, etc.

## Figures and Tables

**Table 1:** Signal transduction pathway of each antibody system.

Antibody System	Signal Transduction Pathway
nGAb	T113, Q123, Y185, A223, Y305, T321, S333, M350, Y360, C455, P483, V512, R554, KG633, N779, F824, Y896, T952, C970, N1007, T1036, G1057, V1070, V1102, V1151, E1201, K1286
nGAb-pep	A4, S12, T20, V30, K45, R61, K106, Y139, S170, S200, A226, A327, G359, S410, T480, S659, F759, I831, T1057, G1070, I1154
GAb	C133, P203, S207, R210, T323, L356, C455, S557, D640, F759, G777, A817, L838, A971, G1005, R1102, T1108, S1152, N1199, K1286
GAb-pep	F98, I105, F134, T171, E186, N209, V406, A558, V766, S840, D857, R973, G1007, S1152, E1201, K1286
GAb-Ag	S14, R107, T113, K206, A230, V324, L353, C453, Q525, E662, S722, Q778, S818, G839, E872, R975, W1004, T1108, P1146
GAb-Ag-pep	Q123, K182, R210, S333, I540, E729, Y780, V820, L838, R1102, S1153, K1286
Common residues	Q123, S207, S326, C455, A558, Q778, D838, R975, R1102, P1146, V1200, K1286

nGAb: non-glycosylated antibody system. nGAb-pep: non-glycosylated antibody–cyclopeptide system. GAb: glycosylated antibody system. GAb-pep: glycosylated antibody–cyclopeptide system. GAb-Ag: glycosylated antibody–antigen system. GAb-Ag-pep: glycosylated antibody–cyclopeptide–antigen system.

**Table 2:** Interaction occupancy analysis at the ligand binding site.

Ligand Bind Site	Interaction Type	Antibody System	Occupancy (%)
Antigen binding site (orthosteric site)	Hydrogen bond	GAb-Ag	4.5
		GAb-Ag-pep	16.7
	Hydrophobic	Gab-Ag	23.7
		Gab-Ag-pep	22.2
Cyclopeptide binding site (allosteric site)	Hydrogen bond	GAb-pep	17.5
		GAb-Ag-pep	67.1
	Hydrophobic	GAb-pep	35.8
		GAb-Ag-pep	46.8

**Table 3:** Interaction count analysis within the entire antibody structure of all systems.

Interaction Type	Antibody System	Min	Max	Average
Hydrogen bond count (pair)	nGAb	146	249	208
	NGAb-pep	162	245	208
	GAb	169	252	213
	Gab-pep	158	260	212
	Gab-Ag	153	251	206
	GAb-Ag-pep	159	248	209
Hydrophobic count (pair)	nGAb	40,544	41,530	41,096
	nGAb-pep	40,604	41,588	41,153
	GAb	40,588	41,590	41,144
	GAb-pep	40,650	41,800	41,256
	GAb-Ag	40,568	41,660	41,126
	GAb-Ag-pep	40,632	41,616	41,142

nGAb: non-glycosylated antibody system. nGAb-pep: non-glycosylated antibody–cyclopeptide system. GAb: glycosylated antibody system. GAb-pep: glycosylated antibody–cyclopeptide system. GAb-Ag: glycosylated antibody–antigen system. GAb-Ag-pep: glycosylated antibody–cyclopeptide–antigen system.

## Data Availability

The original contributions presented in the study are included in the article, further inquiries can be directed to the corresponding authors.

## Abbreviations

Abs, antibodies; ADCC, antibody-dependent cell-mediated cytotoxicity; Acceptor, hydrogen-accepting atom; BCC, Bond charge Correction; CL, light chain constant domain; CH_1_, heavy chain constant domain-1; CH_2_, heavy chain constant domain-2; CH_3_, heavy chain constant domain-3; CG, conjugate gradient; DCCM, dynamic cross-correlation matrix; Donor, hydrogen-donating atom; FcRs, fc receptors; Fab, fragment of antigen-binding; Fc, fragment of crystallizable; FEL, free energy landscape; IgG, Immunoglobulin G; IgG1, Immunoglobulin G-1; NPT, contant particle number/pressure/temperature; PCA, principal component analysis; RMSD, root mean square deviation; RMSF, root mean square fluctuation; Rg, radius of gyration; RDCM, residue distance cross-correlation matrix; RCM, residue contact matrix; RESP, Restrained Electro Static Potential; SD, steepest descent; SPM, shortest path map; VL, light chain variable domain; VH, heavy chain variable domain.
